# Assumptions and Properties of Limiting Pathway Models for Analysis of Epistasis in Complex Traits

**DOI:** 10.1371/journal.pone.0068913

**Published:** 2013-07-30

**Authors:** Sven Stringer, Eske M. Derks, René S. Kahn, William G. Hill, Naomi R. Wray

**Affiliations:** 1 Department of Psychiatry, Amsterdam Medical Center, Amsterdam, The Netherlands; 2 Department of Psychiatry, Rudolf Magnus Institute of Neuroscience, University Medical Center Utrecht, Utrecht, The Netherlands; 3 Queensland Brain Institute, University of Queensland, Brisbane, Australia; 4 Institute of Evolutionary Biology, School of Biological Sciences, University of Edinburgh, Edinburgh, United Kingdom; The Scripps Research Institute, United States of America

## Abstract

For most complex traits, results from genome-wide association studies show that the proportion of the phenotypic variance attributable to the additive effects of individual SNPs, that is, the heritability explained by the SNPs, is substantially less than the estimate of heritability obtained by standard methods using correlations between relatives. This difference has been called the “missing heritability”. One explanation is that heritability estimates from family (including twin) studies are biased upwards. Zuk et al. revisited overestimation of narrow sense heritability from twin studies as a result of confounding with non-additive genetic variance. They propose a limiting pathway (LP) model that generates significant epistatic variation and its simple parametrization provides a convenient way to explore implications of epistasis. They conclude that over-estimation of narrow sense heritability from family data (‘phantom heritability’) may explain an important proportion of missing heritability. We show that for highly heritable quantitative traits large phantom heritability estimates from twin studies are possible only if a large contribution of common environment is assumed. The LP model is underpinned by strong assumptions that are unlikely to hold, including that all contributing pathways have the same mean and variance and are uncorrelated. Here, we relax the assumptions that underlie the LP model to be more biologically plausible. Together with theoretical, empirical, and pragmatic arguments we conclude that in outbred populations the contribution of additive genetic variance is likely to be much more important than the contribution of non-additive variance.

## Introduction

A finding from genome-wide association studies for most complex traits is that the proportion of the phenotypic variance attributable to the additive effects of individual SNPs, i.e. the heritability explained by the SNPs, is substantially less than the estimate of heritability obtained from correlations of relatives using family data. Many explanations for this so-called ‘missing heritability’ have been proposed [1], [2], [3], [4], [5]. One explanation is that heritabilities from family (including twin) studies are overestimated. The problem of bias in heritability estimates has been much discussed in the quantitative genetic literature (e.g., [6], [7], [8], [9]). For example, in the classical twin design of monozygotic (MZ) and dizygotic (DZ) twin pairs, there are only three essential statistics that can be estimated from their phenotypes, namely the MZ resemblance (such as covariance or correlation), the DZ resemblance, and the overall phenotypic variation in the sample. Therefore, only three variance components can be estimated, although many more genetic and non-genetic causal components of variance can be postulated to influence MZ and DZ resemblance. It is well recognized that estimates of heritability may be biased and that it is difficult to separate additive genetic from non-additive genetic components and to separate genetic from common (or shared) family environment components (e.g., [6], [7], [8], [9]). Estimates of heritability using phenotypic data from very distantly related individuals may have trivial bias from epistatic or common environment components compared to additive genetic components but are subject to very large sampling error. Human studies of distantly related individuals of sufficient size are simply not achievable. For disease traits, ascertainment bias in sampling of families for estimation of recurrence risks has long been recognized as a possible cause of inflated estimates of heritability [10], [11]. Lastly, estimates of heritability for disease traits from twin cohorts (collected in restricted clinical settings) may be higher than those estimated from national cohort data, these differences most likely reflecting environmental factors including clinical practice [12].

Recently, Zuk et al. [13] revisited the overestimation of narrow sense (additive) heritability from family studies that could result from confounding with non-additive genetic variance. They referred to the difference between the expected value of the heritability estimated from family data and the ‘true’ heritability as ‘phantom heritability’. To illustrate their arguments, they proposed a limiting pathway (LP) model in which there are 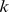 pathway phenotypes, which are unobserved intermediate phenotypes. The phenotypically expressed trait value of an individual is the maximum of the individual pathway values. This model, they suggest, may be representative of biological processes that depend on the rate-limiting value among multiple inputs, ‘such as the levels of components of a molecular complex required in stoichiometric ratios, reactants required in a biochemical pathway, or proteins required for transcription of a gene’. Under their LP model, each pathway phenotype includes only additive genetic effects but, for 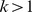, non-additive genetic variance is generated for the expressed phenotype, and so the heritability of this phenotype is less than the expected estimate from a classical twin design analysis. As the magnitude of epistasis depends on 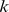, the LP model provides a convenient way to explore the possible contribution of non-additive variation to missing heritability. They use this model to illustrate that over-estimation of heritability from pedigree data may explain an important proportion of missing heritability, but that quantifying this from available data is difficult. They advocate the continuation of association studies but argue that results should be reported acknowledging that heritabilities quoted from family studies may be overestimated. Nonetheless, their results may impact on the design of experiments seeking to identify disease or trait associated variants. It is therefore important to gauge carefully the likely relevance of their results.

Zuk et al. [13] consider their model to be simple and biologically natural. Their model is indeed simple and it usefully explores an epistatic model without needing to define genotypic effects at individual loci, because an infinitesimal model is assumed for each pathway. The pathways are assumed to be genetically independent and to have equal heritability, mean and variance. These are strong assumptions which may not be biologically plausible. For example, in human cells protein concentrations can be correlated and have different variances [14]. As complex traits are affected by many genes, individual genes will typically affect many complex traits [15], [16]. Similarly, a single gene could affect multiple pathways, thereby creating a dependency between the affected pathways.

The purpose of this paper is twofold. First, we show that under the basic LP model, highly heritable quantitative traits produce phantom heritability only if the contribution of common variance is relatively large. Second, we explore the impact of the assumptions underlying the basic LP model. We extend the basic LP model to determine if their conclusions also hold after relaxing some assumptions to obtain a more biologically plausible model. Finally, we interpret the LP model in the context of other published studies.

## Methods

### Notation

Where convention allows, we use Greek symbols for population parameters and Roman for their estimates. In other cases we use a hat ( ∧ ) notation to distinguish estimates from population parameters. Moreover, we use 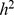 to represent the parameter of narrow sense heritability and 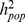 to represent the expected value of the heritability estimated from phenotypic data collected in the population. Here we consider estimation of 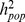 from twin data under the ACE (additive genetic, common environment, unique environment) model, which we denote with 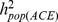. Similarly, we use 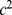 and 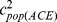 for the parameter and the expected value of its estimate of the proportion of variance attributable to the common environment under the ACE model.

### The classical Twin Model

Like Zuk et al., we explore the LP genetic architecture through the variance components of the classical twin model. Under this model, only three independent parameters can be estimated from sets of MZ and DZ twins. One set of parameters is the phenotypic variance 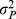, the MZ correlation 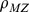 and DZ correlation 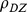. For MZ twins 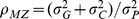 with 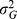 representing the total genetic variance and 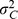 the variance attributable to the common environment. The genetic variance can be broken down into additive 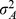 and non-additive components 
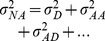
, the sum of all epistatic genetic variance components, with dominance represented by the subscript D. For DZ twins the phenotypic correlation is 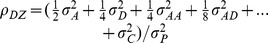. As 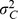 is assumed to be equal in MZ and DZ twins 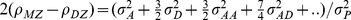 and 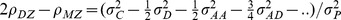. Usually heritability under the ACE model is estimated as 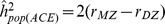 and the proportion of variance attributable to common environmental effects as 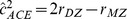.

As well recognized [7], [8], [7,9], 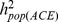 is an upwardly biased estimate of the narrow sense heritability 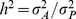. Likewise, when non-additive genetic variance is present 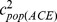 is a downwardly biased estimate of 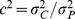, but if 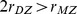 we can conclude that common environment plays a role 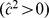. Also recognized in the quantitative genetics literature [7], [6], [9], and concluded by Zuk et al. [13], it is impossible to disentangle the contribution of epistasis and common variance based only on twin data. However, there are bounds on some parameters (and hence their estimates). From the equations provided above and by americanrecognizing that variance components are non-negative, that variance components sum to 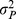, and that MZ and DZ correlations are bounded between 0 and 1, some bounds are 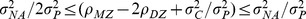, and 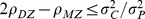. In the absence of dominance and epistasis the lower bound of 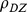 is 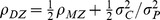. We use these bounds to show in circumstances in which a large contribution from variance from epistasis is possible only if there is a large contribution to the variance from common environment. Whether this is plausible is trait dependent.

### The basic LP Model

In the basic LP model for continuous traits [13], the final observed phenotype 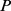 is defined as the maximum (or equivalently the minimum) of 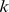 independent intermediate pathway phenotypes, 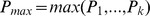. The intermediate phenotypes 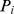 are completely additive, but the final phenotype 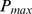 is not if 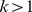. Zuk et al. [13] assumed an infinitesimal model for each pathway, so the basic LP model has three parameters: the number of (additive) genetic pathways 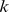, the heritability of each pathway 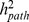 assumed to be constant across pathways, and the proportion of environmental variance 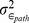 which is common among full siblings (including MZ and DZ twins) 
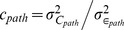
 (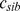 in [13]). For computational convenience the parameter 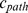 in the LP model is a proportion of the environmental variance to ensure a range between 0 and 1 independent of the value of 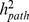. This should not be confused with the previously defined common variance 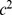 which is proportional to the phenotypic variance.

When 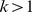, the heritability 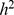 of the expressed phenotype differs from the pathway heritability 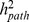, likewise the proportion of environmental variance which is due to common environment in the pathway 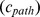 is not necessarily equivalent to the analogous quantity at the final phenotype level 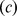. The basic LP model generates no dominance variance, but generates additive × additive variance between loci from different pathways.

### The Extended LP Model

In the basic LP model there are four important assumptions. All pathway phenotypes (i) have the same mean, (ii) the same variance (specifically 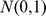), (iii) the same heritability, and (iv) are independent at the pathway level. As these assumptions are unlikely to be upheld in biological systems, we extend the basic LP model by relaxing some of them. In this extended LP model the means, variances and heritabilities may differ, and are defined by, respectively, 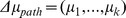, 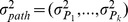 and 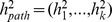. A general correlation matrix could be defined for the genetic relationship between pathways, but for simplicity we assume a uniform genetic correlation between all pathways, 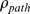 when pathways are all positively correlated. Strong negative correlations between all pathways are not possible in general, so we consider the impact of negative correlations between pathways by dividing the pathways into two equally divided sets which are positively correlated by 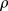 within a set but negatively correlated by 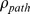 between the sets.

The phenotype of pathway 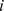 (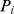) can be partitioned into additive genetic (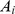) and environmental (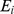) effects, 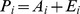. No contribution of common environment is assumed between parents of the same child. The additive genetic variance in pathway 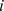 is 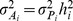, and 
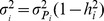
. Illustrating for 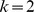, the additive effects for both mothers and fathers are distributed as
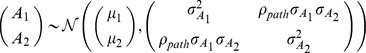



The (unique) environmental (stochastic) effects for parents are assumed to be independent for each pathway and are distributed as



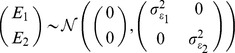



As random mating is assumed, for offspring within a nuclear family the phenotype of pathway 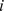 for sibling 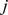 can be partitioned as
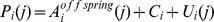
since the additive genetic pathway values of an offspring are distributed with bivariate Mendelian sampling variance about the mean additive genetic values of their parents. 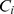 is the environmental effect of pathway 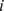 common to all siblings in a family, 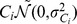, and 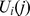 is the environmental effect unique to sibling 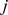, so for 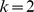,



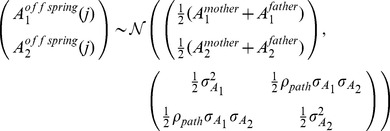



The proportion of environmental variance common for siblings at the pathway level is 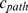 (assumed to be the same for each pathway, and therefore the proportion of variance explained by common environment is 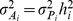 hence 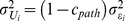 Therefore, the extended LP model is a six parameter model: 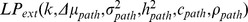. For example, the limiting pathway model 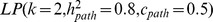 could be modeled with the extended LP model as 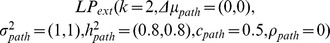. Zuk et al. [13] showed that the narrow-sense heritability of the observed phenotype in the population is 
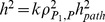
, where 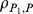 is the correlation between the first pathway phenotype and the final phenotype. This definition assumes exchangeability and independence of the intermediate pathway phenotypes 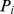. Under the extended LP model, the pathway phenotypes are non-exchangeable and correlated. As the additive model assumes 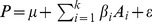, we can estimate the pathway coefficients 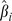 by regressing final phenotype 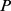 on the pathway genetic values 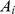. The heritability estimate is a function of the regression coefficients, the additive values and the phenotype variance: 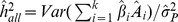. Unbiased estimates of 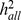 and 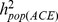 are reported as the mean of 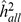 and 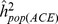 across simulation replicates. As defined in Zuk et al. [13], the phantom heritability is 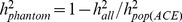.

### Simulation

For all simulations, we generated 50 independent samples of 100,000 families. Each family comprised two parents, an offspring, its MZ twin and its DZ twin. The phenotype of a parent for pathway 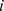 (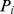) was simulated as 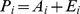. With random mating of parents, the phenotype of an offspring 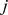 for pathway 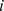 was simulated as
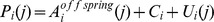
with 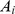, 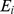, 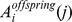, 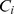, and 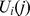 drawn from their respective multivariate distributions. For monozygotic twins 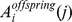 = 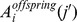. In all simulations, unless stated otherwise, the following parameters were used: 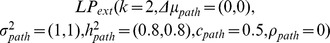. From the final phenotypes of the offspring, the twin correlations 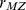 and 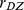 were calculated, resulting in a heritability based on the ACE model of 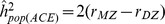 and phantom heritability 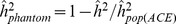. The reported 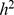, 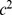, 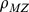, 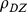, and 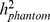 are means across 50 simulation replicates and hence unbiased.

We first performed simulations to study the implications of the basic LP model with respect to common environmental effects. To explore bounds on variance components we simulated a range of basic LP models 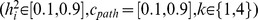, and calculated 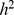 and 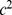 for each model. From each simulation we estimated 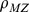 and 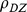 and plotted 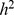 and 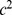 as a function of 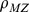 and 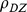.

Subsequently we performed five simulations to study the effect of differences in (i) pathway mean, (ii) variance, and (iii) heritability on phantom heritability, and (iv & v) the effect of correlations between pathways on phantom heritability in the extended LP model. Simulations (i) to (iii) comprise a series of two-pathway models in each of which one parameter was changed: (i) the difference in pathway mean (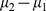). These values are in standard deviation units since 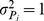. (ii) The pathway variances differed, 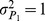 and 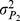 was varied. (iii) The pathway heritabilities differed, 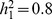 and 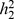 was varied. (iv) In this case a multiple pathway model was simulated with no common environment effects (i.e., 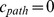), in which both the pathway correlations 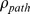 and number of pathways 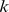 were varied. (v) As (iv), but with 10% of the variance of each pathway attributed to common environment (i.e.,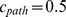 when 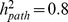).

Finally, to illustrate the extended LP model, we chose three continuous traits with different ACE-based heritability estimates from studies found in a recent twin research review paper [17]: (i) height in Danish male twins 
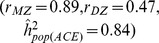
 [18], (ii) triglyceride levels in blood in Swedish female twins from 20–29 years old 
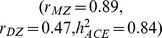
 [19], and (iii) high-fat dairy intake in UK male and female twins adjusted for age 
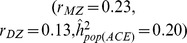
 [20]. Based on these observed values and for different combinations of 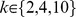 and 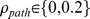, we report the estimates 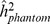, 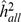, and t 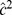. As 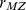 and 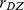 are not model parameters but model outputs, the input parameters 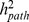 and 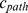 were chosen such that estimated 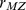 and 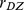, based on the median of 50 simulations, reflected the observed values.

## Results

### Exploring Bounds of Variance Components

**Figure 1:**
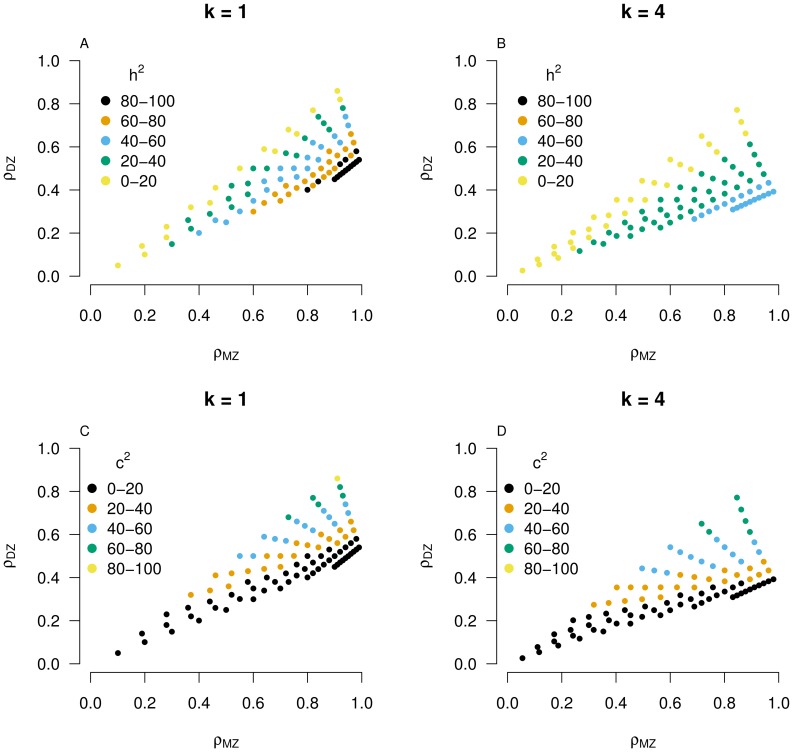
Mean narrow-sense heritability (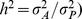 (panels A and B) and proportion of phenotypic variance which is common for siblings 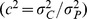 (panels C and D) across 50 simulation replicates color coded as function of MZ/DZ correlation and number of pathways (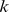) under the basic LP model. Given a combination of MZ/DZ correlations, a decrease in narrow-sense heritability (i.e., as k grows), implies an increase in contribution of common environment.

Although it is impossible to disentangle the contribution of non-additive genetic variance and common variance from twin data, there are some bounds on these parameters as illustrated in Figure 1, generated under the basic LP model, which is shown to yield more extreme non-additive genetic variance than many extended LP models. Each point in Figure 1 represents 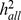 or 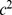 as a function of 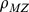, 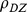 and 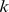.

In comparisons of the left 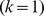 with the right panels 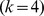, each combination of 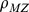 and 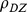 values is consistent with multiple basic LP models. In other words, the number of pathways cannot be derived from a pair of 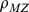 and 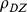 values alone. As expected, for any 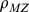 and 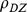 combination, the non-additive variance increases with the number of pathways, resulting in a lower narrow-sense heritability (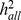) estimate for an epistatic model 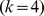 compared to the additive model 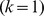 (panels A vs B). However, the contribution of environmental variance which is common for siblings 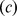 increases as well (panels C vs D). Therefore, as 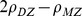 increases for complex traits, important contributions from non-additive variance can be achieved only if accompanied by high 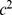. For example, if 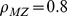 and 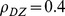, an additive model 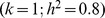 implies no contribution of common variance 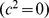, whereas an highly epistatic model (e.g., 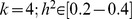) is consistent only with 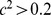. More generally, for highly heritable traits 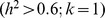 a large amount of epistasis 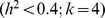 is consistent only with 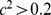. However, if 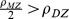 then substantial phantom heritability need not be accompanied by large 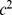.

### Properties of the Extended LP Model

**Figure 2:**
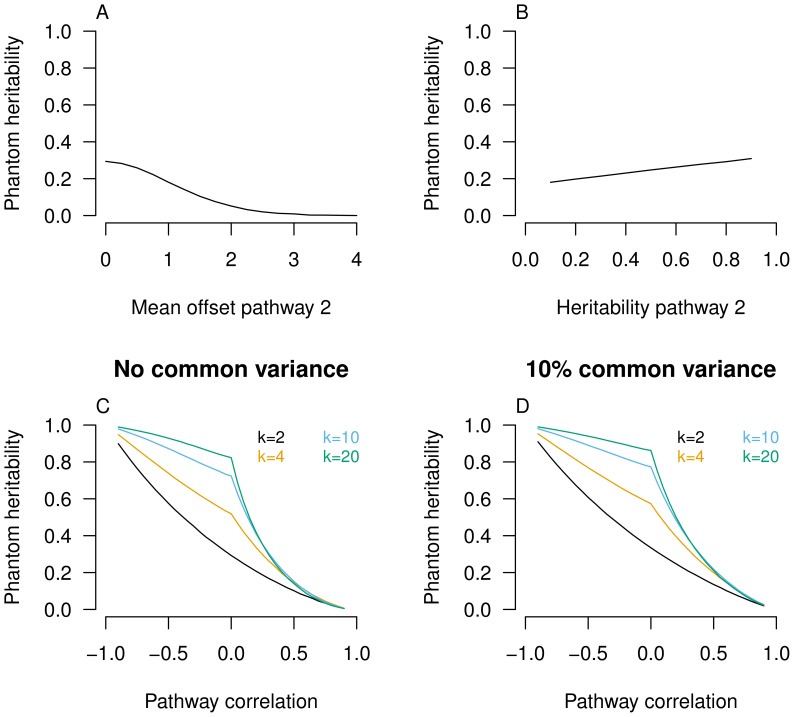
Phantom heritability under the extended LP model as a function of (A) differences in mean (sd unit) of two pathway phenotypes, (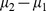) (B) changes in 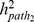 while 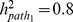, (C) pathway correlations for different number of pathways, and (D) pathway correlations for different numbers of pathways assuming a total contribution of common environment of 10% for each pathway phenotype. Unless stated otherwise, number of pathways 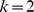, contribution of common family environment is zero (i.e., 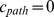), pathway heritability 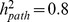, pathway correlations 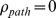.

Simulation results of the extended LP model are reported in Figure 2 for the effect of different parameters on the phantom heritability: (i) As the offset in mean between the two pathways increases, the phantom heritability decreases (panel A). One standard deviation difference in mean between pathway phenotypes (if 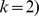 approximately halves the phantom heritability. Clearly, as differences in offset become large, some pathways contribute little to the final phenotype, effectively decreasing the number of contributing pathways and hence the amount of epistasis. (ii) Differences in phenotypic variance between two pathways had no effect on phantom heritability (result not shown), because large phenotypic variance not only increases the probability of producing a maximum value, but also increases the probability of producing a minimum value. Across individuals both pathways contribute equally to the observed phenotype, but the mean and variance of the observed phenotype increases. Although the correlation between the final phenotype and the pathway phenotypes is higher for the pathway with the higher variance, the variance of the observed phenotype increases proportionally with the ratio of the two pathway variances, resulting in a constant heritability. (iii) As panel B shows, differences in heritability between pathway phenotypes have only a marginal effect on phantom heritability. (iv and v) Correlations between pathways affect the phantom heritability significantly (panels C and D). Positive correlations between pathways effectively limit the amount of epistasis, resulting in less phantom heritability. The larger the number of pathways, the larger the phantom heritability reduction (for any given pathway correlation). As the correlation between pathways approaches 1, the model approaches an additive single pathway model. This holds irrespective of the amount of common variance assumed, although common variance increases the phantom heritability slightly (panels D vs C). These results show that relaxing the assumptions of equal mean and uncorrelated pathways can substantially reduce the amount of phantom heritability. In contrast, negative correlations increase the amount of epistasis even if only one out of the 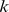 pathways is negatively correlated to the remaining positively correlated pathways, although the relative impact decreases as 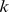 increases (panels C and D).

### Illustration for Three Traits

Table 1 shows the implications of the (extended) LP model for three continuous traits with increasing estimated heritability: high-fat dairy intake 
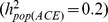
, triglyceride levels in blood 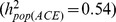, and height 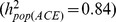. The table illustrates two important points. First, assuming a larger amount of epistasis (i.e., larger 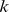), not only implies increased phantom heritability and decreased narrow-sense heritability, but also implies a larger contribution of common variance. Second, assuming positive dependence between pathways 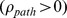 reduces the amount of epistasis. In other words, increasing the number of pathways has less effect on phantom heritability, narrow-sense heritability, and the contribution of common variance, compared to a model with 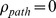. Especially for traits with a large estimated ACE heritability, a high phantom heritability 
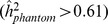
 is only compatible with a scenario in which the percentage of common variance is high 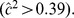.

**Table 1:** Phantom heritability 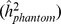, narrow-sense heritability 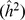 and percentage of common variance 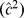 for three traits assuming varying number of pathways 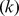 and pathway correlations 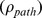.

Trait	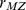	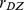	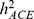	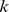	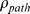	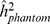	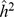	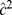
**High-fat dairy intake**	**0.23**	**0.13**	**0.20**	2	0	0.15	0.17	0.04
				4	0	0.30	0.14	0.06
				10	0	0.56	0.09	0.10
				2	0.2	0.05	0.19	0.03
				4	0.2	0.13	0.17	0.04
				10	0.2	0.22	0.16	0.06
**Triglyceride levels in blood**	**0.55**	**0.28**	**0.54**	2	0	0.25	0.41	0.07
				4	0	0.49	0.28	0.16
				10	0	0.74	0.14	0.29
				2	0.2	0.16	0.45	0.05
				4	0.2	0.28	0.39	0.09
				10	0.2	0.38	0.33	0.15
**Height**	**0.89**	**0.47**	**0.84**	2	0	0.35	0.55	0.20
				4	0	0.61	0.33	0.39
				10	0	0.82	0.15	0.61
				2	0.2	0.26	0.62	0.17
				4	0.2	0.43	0.48	0.30
				10	0.2	0.56	0.37	0.43

Illustrated for observed values of the estimated heritability 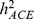 and underlying 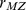 and 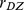, assuming a larger number of pathways implies higher phantom heritability, lower narrow-sense heritability, but also a larger contribution of common variance. Higher pathway correlations reduce these effects.

Nonetheless, in some scenarios important phantom heritability is expected with negligible 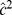, for example when *k* = 2, 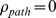 we estimate 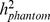 to be 0.25 and 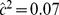. We note that we selected examples with 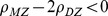 to illustrate potential implications of the LP model. Hill et al. [21] reported an empirical distribution of 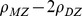 distributed around zero, with interpretation that the distribution reflected sampling variance given the often small sample size. However, direct interpretation of the point estimates suggests that, since 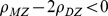 in ^∼^50% of cases, in these cases substantial phantom heritability could be present in the context of zero or weak common environmental variance.

## Discussion

### Bounds of Variance Components in Twin Studies

Using the basic LP model we explored constraints on combinations of parameters. For additive models (Figures 1A and 1C) all combinations of 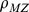 and 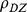 lie in a region bounded by 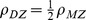 (if 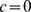) and 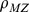 = 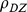 (if 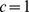). For epistatic models (Figures 1B and 1D) the bounds are evident from the wedge shape of permissible combinations of 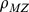 and 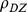 in Figure 1. They show that when 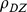
>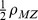, substantial non-additive genetic variance can be accompanied only by unreasonably high 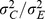. This implies that, at least when 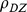
>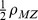 an underlying additive model is more plausible than a highly epistatic architecture. Specifically, as Table 1 illustrates, in highly heritable traits with a small contribution of common variance, phantom heritability is likely to be small.

As noted by Zuk et al. [13] in their supplementary information, the amount of phantom heritability estimated depends on the method of estimation of 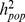. The expected heritability estimate from regression of offspring phenotype on mid-parental phenotype 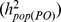 is less than 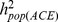 under the 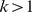 basic LP model. Other factors could also contribute to differences between 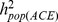 and 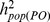 such as dominance and greater common environment of sibling compared to filial relations. Despite this, empirical observation ([7] pp. 172–173) does not, in general, suggest large differences between 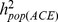 and 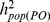, which is not consistent with an important role for phantom heritability (although sampling variation about estimates make it difficult to draw strong conclusions). Deconfounding of genetic and common environmental variance is possible, for the most part, by use of adopted away relatives. Very different estimates of correlations between adopted away siblings and those raised together is expected if phantom heritability is important, but adoption studies tend to support genetic estimates from twin studies [22].

### The Extended LP Model

Zuk et al. proposed a simple and elegant model that allows exploration of the impact of epistasis on estimates of heritability without needing to define epistasis between individual loci. In fact, the basic LP model is a special case of the optimum pathway model proposed by Sewall Wright in 1935 [23], in which the expressed phenotype is the pathway value closest to a defined optimum, which could be, for example, the mean or median, rather than the maximum. These models include the additive model as a special case, but produce different amounts of epistasis as the number of pathways increases. Indeed any non-linear transformation of an additive genetic model, even the infinitesimal model, leads to non-additive variation; but Zuk et al. show that the basic LP model generates a phenotypic distribution close to normal, particularly when *k* is small. Under the basic LP model all pathway phenotypes have the same distribution and pathway heritabilities and pathways are uncorrelated. Biologically, these are very strong assumptions, not least since they invoke the infinitesimal model that implies independent contributions from many genomic sites in each pathway, and so we extended the basic LP model to allow correlated pathway phenotypes with different distributions and pathway heritabilities. Phantom heritability was little affected by differences in variance and heritability between pathways. However, differences in mean phenotype and the presence of positive correlations between pathways can decrease the phantom heritability considerably, and negative correlations increase it. Our results show how the predicted importance of phantom heritability depends on implicit model assumptions, such thatthe problem of phantom heritability could be overstated.Drawing inferences about epistasis from the LP model.

There is much debate about the relative importance of non-additive versus additive genetic variance [24], [25], [26], [21], [27], summarized by Crow [28]. Central to the debate is that mutational studies demonstrate the ubiquity of epistasis in the classical sense, because genes interact in hierarchical systems to generate biological function [26]. However, in quantitative genetics it is the residual variation segregating in populations that determines differences amongst individuals not overall biological function [26]. Fisher suggested that epistasis was not important because usually there would be some scale transformation of phenotypic values to generate additive effects [26]. Indeed, this is the basis of models of complex disease where non-additivity on the observed scale can be transformed to an underlying additive scale. Furthermore, under mutation drift (neutral) models a high proportion of genetic variants are at frequencies near 0 or 1, so the presence of substantial epistatic interactions at the level of gene effects does not in itself generate appreciable epistatic variance, and contributions from epistatic interactions are detected as additive variance [28], [21]. These arguments are further strengthened under models that consider selection against mutations deleterious for fitness with pleiotropic effects on quantitative traits [29] as the proportion of variants with frequencies near 0 or 1 is even higher. Zuk et al. [13] incorrectly state in their Supplementary Information that the derivation in Hill et al. [21] applies only to pairs of loci, whereas in fact these wereused for illustration, and the argument holds for multilocus epistasis. The elegance of the LP model of Zuk et al. [13] is that it is parametrized in terms of variances and so does not depend on the allele frequency distribution. Zuk et al. [13] (supplement page 45) counter Hill et al's analysis by arguing that most genetic variants contributing to complex traits cannot be at extreme frequencies because these would generate little variance. They illustrate with a two-locus example (their Supplementary Figure 9), but it shows a steep increase in total genetic variance from minor allele frequency of 0 to 0.1, nearing its maximum for minor allele frequency 0.1, where additive × additive variance accounts for only 8% of the genetic variance. Furthermore, because the distribution of heterozygosity is approximately uniform over 0 to 1 under the neutral mutation drift model, all frequencies are expected to contribute approximately equally to the variance under an additive model. Empirical results also suggest that epistasis can generates little epistatic variance. For example, although many substantial epistatic effects have been detected for bristle number in Drosophila [30], bristle number expresses mostly additive variance in populations [16]. At face value these results may seem to be contradicted by recent results of the Drosophila Genetics Research Panel (DGRP) entitled “Epistasis dominates the genetic architecture of Drosophila quantitative traits” [31]. They reported data are from a GWAS undertaken on the 168 DGRP lines [32] and on gene frequency differences between pools of lines scoring high and low for phenotypes following an advanced intercross (70 generations) from 40 of the DGRP lines [31]. They found no overlap of SNP associated effects between the two analyses, which they interpreted as presence of epistasis. However, the limited number of DGRP lines are underpowered for association analysis and show long range LD so effects of distantly located QTL are confounded and are less likely to match those found in the intercross study. Also, as the authors [31] note: “In fact, variation induced by all of the epistatic interactions identified in the present study could be largely explained by the marginal additive effects at the trait-associated loci”.

### Limiting Pathways in Context

The LP model was justified (Zuk et al., p1193 [13]) without reference as: “Here we show that simple and plausible models can give rise to substantial phantom heritability. Biological processes often depend on the rate-limiting value among multiple inputs, such as the levels of components of a molecular complex required in stoichiometric ratios, reactants required in a biochemical pathway, or proteins required for transcription of a gene.” For biochemical pathways, at least, metabolic control theory has shown that ‘rate limiting steps’ are not a relevant concept, for rate of flux is a continuous function of activities at multiple stages of the pathway [33]. In a recent review Suarez and Moyes stated “The days have long passed when it was simply assumed that enzymes possessing allosteric regulatory properties were ‘rate-limiting’ [34]. It is now recognized that control of pathway flux is often distributed among many enzymes.” And Fell’s well-cited review [35] concludes “whatever criticisms might be made about any one of the experimental studies, it is significant that none have provided support for the existence of unique ‘rate-limiting’ enzymes in pathways.” In quantitative genetic analysis of models of such pathways, it has been shown that a substantial proportion of the variance is additive [36].

The LP model was proposed to explain “missing heritability” in complex traits. Methods are now available to estimate variance attributable to all common genotyped SNPs rather than those identified as significant [37], [38]. Simulations conducted under the LP model demonstrate that estimates of additive variance attributable to SNPs calculated using GCTA [39] are unbiased and not inflated by epistasis [40]. Applications of these methods to real data show that at least 40% of heritability estimated from family studies remains unexplained [41]. The number of associated common variants detected has increased with sample size [41] e.g., from 9 to 140 for Crohn’s Disease as case sample size increased from 2000 [42] to 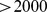 [43]. The implication is that, to date, studies have been underpowered to detect common variants of realistic effect sizes, but that many exist, given that rare variants are much more prevalent, and that a very large number of rare variants also contribute exist which individually explain little variance but their cumulative contribution may be important. Collection of empirical data to test an additive only model is unlikely to be achievable in humans. In yeast, an elegant study designed to explore contributions of variance from different sources found substantial epistatic variance (median of 30%) for some of the 46 traits studied [44]. However, its relevance to human populations is limited, since all gene frequencies were one half (two-way cross design), conditions under which epistasis is likely to be maximized [21], [28]. More relevant insight may be gained from outbred species. For example, in dairy cattle heritability and SNP associated effects are estimated from large numbers of half-sib daughters born, raised and milked at different farms. Therefore, their estimates are unlikely to be confounded with non-additive genetic or shared environmental effects [45]. For milk yield 79% (s.e. 5%) of the additive genetic variance is captured by SNPs [46]. That there is so little missing heritability can be explained by the smaller effective population size leading to longer linkage disequilibrium (LD) blocks than in humans and hence even rare alleles can be predicted by multiple SNPs. Traits that could reasonably be assumed to be under strong natural selection (so that very rare variants play an important role), such as fertility, have lower heritability (40%) and greater missing heritability (55% explained by common SNPs fitted together) [46]. The simplest explanation of why not all variance is explained by the SNPs is that even in livestock some causal variants are rare and in low LD with the SNPs. These results provide evidence that (when 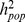 is estimated accurately) additive effects can explain the majority of observed variance in a complex trait in an outbred population.

### Disease Traits

Zuk et al. [13] expressed phantom heritability as 
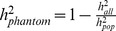
. For quantitative traits 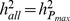, where 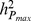
is the proportion of variance in 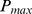 attributable to additive genetic factors. For disease traits they considered a liability threshold model, but did not assume disease to occur when the liability phenotype 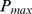 exceeds the threshold truncated by the proportion 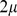, but instead defined disease to occur when a pathway phenotype exceeds the threshold truncated by the proportion 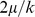, generating a total proportion, 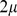, of affected individuals when summed over all 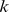 pathways. This definition implies additional non-additive genetic variance, i.e., 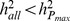. For example, using the 3-pathway model for Crohn’s Disease [13] with 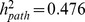 and 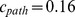 generates 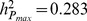, but 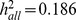 for 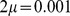. Under the extended LP model we showed for quantitative traits that results for a multiple pathway model converged to a single pathway model for positively correlated pathways; qualitatively this result also holds for disease traits.

The LP model was justified by Zuk et al as limiting pathways in a biological and biochemical sense. However, the same methodological approach could represent a heterogeneity model, generating a different interpretation of results. Under the LP model the final phenotype is considered the “true” phenotype and the non-additive genetic variance 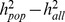 is real. In contrast, under a heterogeneity model, the pathways are the true phenotypes but inadequacies in phenotyping cause an inability to distinguish between biologically different classes of the observed disease. Hence, under a heterogeneity model, the measurable additive genetic variance 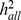 may be much less than true additive variance of each subtype, but mostly 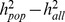 could be viewed as “phantom non-additive genetic variance”, since the non-additive genetic variance results only from incorrectly treating multiple phenotypes as a single trait. In common complex genetic disease there have been notable advances in separation of diseases that originally were considered a single diagnostic class, e.g., diabetes, rheumatoid arthritis, breast cancer. Dilution of allelic effect size is a consequence of phenotypic heterogeneity in genetic association studies. For example, differentiation of breast cancer into ER-positive and ER-negative cancers has identified associated loci not possible from combining the case cohorts [47]. In psychiatric nosology it has long been recognized that diagnostic classes are likely to overarch heterogeneous etiology, recently explored in light of results from genomic studies [48]. Indeed, one motivation of genomic studies is to allow genetically informed nosology.

### Conclusion

The results of Zuk et al. [13] provide a timely reminder of the well-recognized limitations of analyses based on twin and family data, which are often underpowered to separate additive genetic from common environmental effects [49] and non-additive effects. The (extended) LP model provides a useful framework to explore the possible contribution of non-additive genetic variance to complex traits. An important role for non-additive genetic action is attractive because gene interactions are ubiquitous at the functional level, yet this does not necessarily translate to important epistatic variance over and above variance detected as additive effects. For disease traits, empirical data can only be explained by non-additivity on the disease scale, but such non-additivity can be explained by scale transformations without needing to invoke more complex models. Using the framework of the extended LP model, and together with theoretical, empirical, and pragmatic arguments we conclude that although contributions from non-additive variance may be commonplace in complex traits, the contribution of additive genetic variance is likely to be much more important than that of non-additive variance. Ultimately, only empirical results can provide a satisfactory conclusion to the debate of missing heritability, but these may be elusive. Larger sample sizes should afford the power to identify common variants of smaller effect size and two-locus interactions. However, the heavy penalty of multiple testing will not allow exploration of higher order epistatic interactions implied by the LP model. Likewise, large sample sizes are unlikely to identify rare causal variants of small effect, since rare variants are likely to be population specific and large sample sizes from homogenous ethnic groups simply may not exist. Zuk et al. [13] suggest a methodology for estimation of 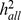, but the required cohorts (large and from isolated populations) are also difficult to achieve. For disease traits the most tractable approach may be collection of large, informatively phenotyped cohorts to provide the building blocks that may allow clustering of cases based of combinations of genetic risk variants to be mapped onto phenotypic heterogeneity.
